# The addition of computed tomography (CT) findings maintains the predictive performance of an existing radiograph-based donor lung acceptability score

**DOI:** 10.1016/j.jtcvs.2026.01.035

**Published:** 2026-02-17

**Authors:** Yun Zhu Bai, Charles R. Liu, Zhizhou Yang, Yan Yan, Su-Hsin Chang, Anjana Delhi, Chad A. Witt, Rodrigo Vazquez Guillamet, Derek E. Byers, Gary F. Marklin, Bruce Nicely, Michael Harmon, Andrew J. Bierhals, Matthew G. Hartwig, Whitney S. Brandt, Ruben G. Nava, Bryan F. Meyers, Benjamin D. Kozower, Michael K. Pasque, G. Alexander Patterson, Daniel Kreisel, Varun Puri

**Affiliations:** a Division of Cardiothoracic Surgery, Washington University School of Medicine, St Louis, Mo; b Department of Surgery, Massachusetts General Hospital, Boston, Mass; c Division of Public Health Sciences, Department of Surgery, Washington University School of Medicine, St Louis, Mo; d Division of Pulmonary and Critical Care Medicine, Department of Medicine, Washington University School of Medicine, St Louis, Mo; e Mid-America Transplant, St Louis, Mo; f Gift of Life Michigan, Ann Arbor, Mich; g Gift of Hope Organ & Tissue Donor Network, Itasca, Ill; h Mallinckrodt Institute of Radiology, Washington University School of Medicine, St Louis, Mo; i Division of Cardiovascular and Thoracic Surgery, Department of Surgery, Duke University Medical Center, Durham, NC.; j Department of Pathology and Immunology, Washington University School of Medicine, St Louis, Mo

**Keywords:** lung transplantation, brain-dead donor, chest CT, chest radiograph

## Abstract

**Objective:**

The use of lungs from brain-dead donors is low partly as a result of the lack of reliable donor assessment criteria. The validated Lung Donor (LUNDON) score predicts lung acceptance for transplantation by using 9 clinically relevant variables, including the presence of an abnormality on radiograph of the chest. Because most organ donor evaluations now include routine computed tomography (CT) of the chest, we aimed to assess whether the addition of CT findings impacts the LUNDON model’s performance.

**Methods:**

Data including CT findings were collected for adult brain-dead donors from 3 organ procurement organizations from 2014 to 2020. The primary outcome was lung acceptance for transplantation. We collated all CT findings into a weighted CT composite score, with greater scores representing more CT abnormalities, and calculated the score for each donor.

**Results:**

The lung acceptance rate was 40.4% among 2454 donors with CTs of the chest and 22.3% among 1980 donors without CTs of the chest. Emphysema, pulmonary edema, and traumatic lung injury on CT were associated with a lower likelihood lung acceptance. The LUNDON model’s performance was comparable between use of the original radiograph of the chest variable, the CT composite score, or both variables together (C-statistics 0.883, 0.887, 0.890, respectively). All 3 iterations of the model were predictive of 1-year graft survival.

**Conclusions:**

Undergoing CT was independently associated with donor lung acceptance. The incorporation of highly granular findings from CT of the chest to the previously established LUNDON model maintained, but did not meaningfully improve, its excellent baseline ability to predict lung use and its association with graft survival. (J Thorac Cardiovasc Surg 2026;171:1341–50)

## CENTRAL MESSAGE

The addition of a composite score based on chest CT findings maintained the Lung Donor (LUNDON) acceptability score’s predictive performance of braindead donor lung use.

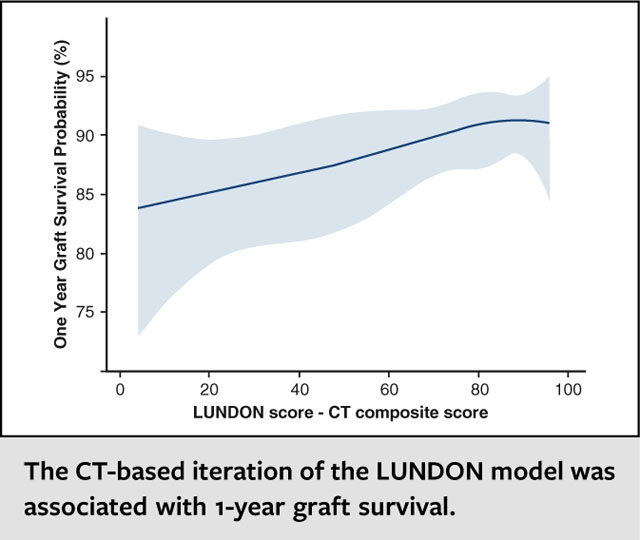

Lung transplantation is the definitive treatment of end-stage lung disease, but organ demand outpaces donor supply, leading to approximately 15% of patients on the waitlist dying or becoming delisted.^1,2^ This issue is further exacerbated by the low use of available donor lungs, a pattern partly attributable to overly stringent donor assessment criteria.^3,4^ To enhance donor lung assessment practices, our group has developed and validated a novel, highly predictive scoring system called the Lung Donor (LUNDON) acceptability score or model.^5^

The LUNDON model predicts lung acceptance for transplantation by using 9 donor variables: age, cause of death, creatinine, oxygenation, bacteremia, history of smoking, history of myocardial infarction, history of cardiac arrest, and the presence an abnormality on a radiograph of the chest (CXR). The LUNDON model is distinguished from other donor acceptability algorithms by its generalizability, because it was derived using data from a large national cohort from the Scientific Registry of Transplant Recipients (SRTR). Furthermore, unlike other donor acceptability algorithms, which include recipient characteristics and outcomes, the LUNDON model is based strictly on donor criteria.^5–10^ This allows the model to accurately predict lung acceptance for transplantation while accounting for organs that were rejected. Nonetheless, we have also demonstrated that a greater LUNDON score is predictive of graft survival in recipients with lung allocation scores >70, whereas lower and higher LUNDON scores were associated with similar rates of graft survival in recipients with lung allocation scores ≤50.^11^ Therefore, the LUNDON model is positioned to potentially increase use of donor lungs by guiding optimal donor management practices and organ assessment by transplant centers in the matching process.

The use of CXR findings as the sole imaging criteria in the LUNDON model is consistent with standard donor lung-evaluation parameters outlined by the International Society of Heart and Lung Transplantation.^4^ CXR may be used to identify lung abnormalities and donor-recipient size matching.^12^ Ideal donor criteria include normal CXR findings, but donor lungs with abnormal CXRs are frequently accepted, given the shortage of available organs.^13^ In the modern era of lung transplantation, computed tomography (CT) of the chest is increasingly used in donor evaluation and, compared with CXR, offers greater diagnostic sensitivity and accuracy.^14,15^ Several institutional studies have shown that CT of the chest may independently aid in the decision to accept or reject donor lungs and that CT of the chest volumetry may improve the accuracy of donor-recipient size matching.^2,4,16–18^ Although these studies have described imaging characteristics that predict lung acceptance, a holistic composite score of CT abnormalities has not been previously developed.

Given this gap in the existing literature, we aimed to develop a CT composite score based on standard assessment of clinically relevant CT abnormalities and test our hypothesis that the composite score could predict lung acceptance among brain-dead donors. We then investigated whether the addition of the CT composite score to the LUNDON model would improve its performance in predicting lung acceptance.

## METHODS

Brain-dead donors managed at 3 US organ procurement organizations (OPOs) between January 2014 and June 2020 were included in the study cohort. Standard demographic, clinical, and laboratory data for these donors were obtained via a special data request from the SRTR, and additional site-specific clinical and radiographic data were collected from individual OPOs with appropriate permissions. Exclusion criteria included age younger than 18 years and an absence of CTs of the chest obtained. Recipient outcome data were obtained from the SRTR. The Washington University in St Louis Human Research Protection Office reviewed the project and determined that it was exempt from institutional review board review.

In all cases, the donor CT images included in this study were interpreted by board-certified radiologists as a part of donor management. Subsequently, highly granular data and severity of all radiographic abnormalities for each CT were manually abstracted from the radiologists’ reports by study team members using a standardized format. At the time of data abstraction, the team member(s) extracting these data did not have information regarding the outcome of the donor lungs and recipient outcomes, thereby blinding them to the outcomes of interest in our analysis. With input from a panel of experts including lung transplant surgeons, transplant pulmonologists, thoracic radiologists, and statisticians, the following CT findings were categorized as binary (yes vs no): pulmonary hypertension and/or right heart strain, emphysema, interstitial lung disease (ILD), pulmonary embolism, pulmonary edema, and nodules that were Lung CT Screening Reporting & Data System (Lung-RADS) category 3 or greater (which included ground-glass opacities); whereas the following CT findings were categorized by severity level: traumatic lung injury (none vs segmental or less vs lobar or greater), atelectasis (none vs segmental or less vs lobar or greater), pneumonia (none vs segmental or less vs lobar or greater), and pleural effusion (none vs small vs moderate/large). In the event that multiple CTs were performed for a donor, the final CT performed before organ procurement was used for analysis.

The primary outcome measure was lung acceptance, which was defined as at least 1 lung accepted with the intent to transplant. We first performed univariate comparisons to assess the baseline characteristics of our cohort who had at least 1 lung accepted versus those who did not. In addition, to identify possible selection bias, we evaluated the baseline characteristics of donors who underwent CT versus those who did not, using the Student *t* test for continuous variables and the χ^2^ test for categorical variables. Next, to understand the impact of CT evaluation on donor assessment, all CT findings for each donor were collated into one easy-to-understand, weighted “CT composite score” variable. First, beta coefficients of CT variables were obtained in a multivariable logistic regression model for lung acceptance, where covariates were also included based on clinical relevance and statistical significance on preliminary univariable analysis. Subsequently, the beta coefficients for each CT finding, which represent their effect on lung acceptance, were summed and were individually assigned a weight on the basis of this sum; a CT score composite can then be calculated using the weights (Box 1). A greater CT composite score was associated with a greater number of donor CT abnormalities.

To examine whether the addition of CT findings improved the performance of the existing and previously validated LUNDON model^5^ in predicting lung acceptance, we tested 3 models to predict lung use: (1) the original LUNDON model with the binary CXR variable (normal vs abnormal) alone (“CXR model”), (2) a LUNDON model with the CT composite score substituting the CXR variable (“CT model”), and (3) a combined LUNDON model with the binary CXR variable and the CT composite score (“combined model”). In order to compare model performance, the CXR model was calibrated to the current study cohort using a new intercept while keeping the exact transformation and coefficients for all variables. For the CT model, the coefficient for the CXR variable was removed and the rest of the original LUNDON model was kept as an offset while the CT composite score was added to the model. The combined model was similarly fitted with the entire original CXR model kept in an offset while the CT composite score was introduced as a new variable. Model performance was compared using the C-statistic (a greater value indicates better discrimination) and the Brier score (a lower value indicates better model calibration).

Finally, we sought to evaluate the relationships between each of the 3 LUNDON models and 1-year graft survival rate (including graft failure and recipient all-cause mortality). The available recipient data were matched with donor data using United Network for Organ Sharing identification numbers. Patients who underwent retransplant were excluded from the analysis. The relationships were then plotted using restricted cubic spline (RCS) curves with 4 knots at fifth, 35th, 65th, and 95th percentiles.

Normality was assumed for statistical analyses. This was done so in accordance with the central limit theorem, which states a random sample of a given population will have an approximately normal distribution as the sample’s size increases (usually n ≥ 30), regardless of the distribution of the population.^19^

## RESULTS

**FIGURE 1.**
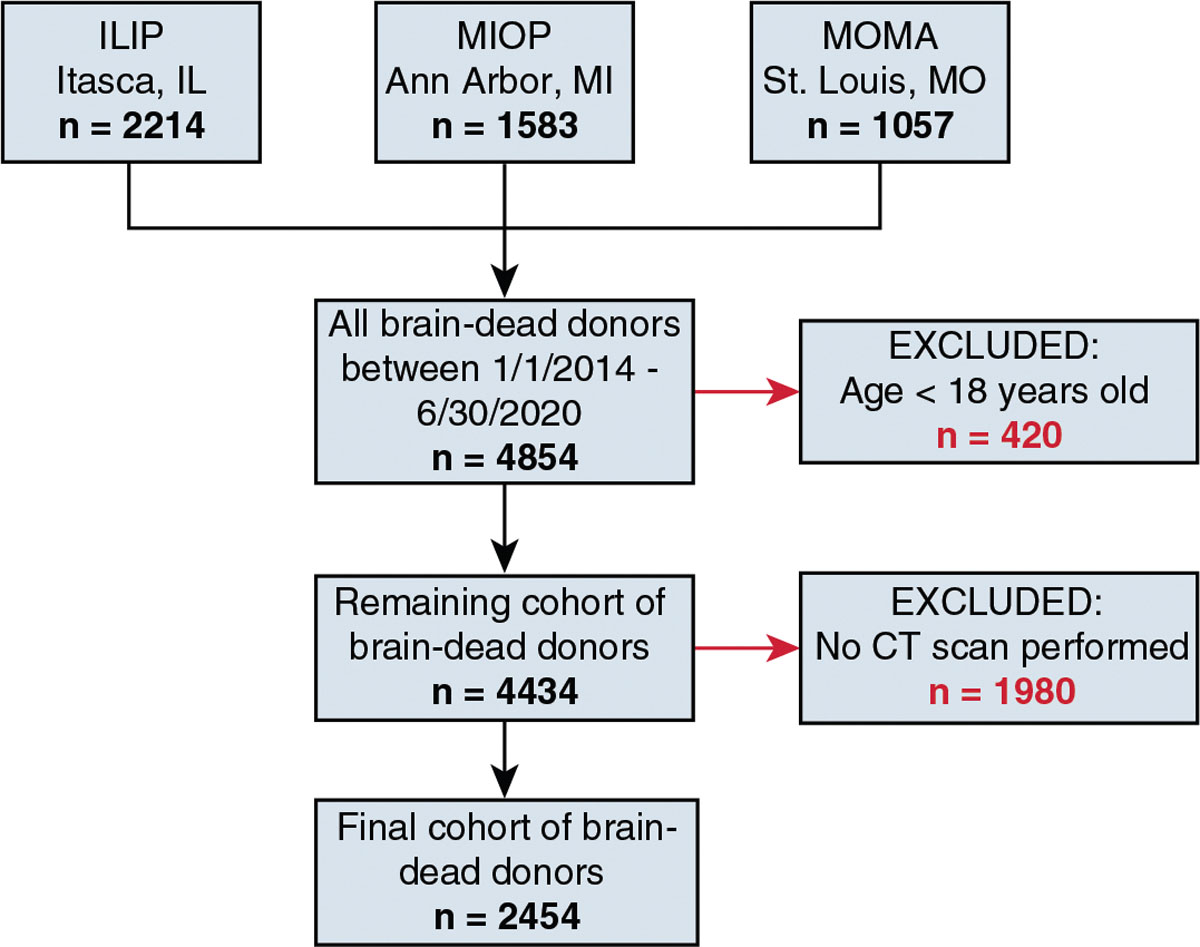
Consort diagram of final donor cohort. *ILIP*, Gift of Hope Organ & Tissue Donor Network; *MIOP*, Gift of Life Michigan; *MOMA*, Mid-America Transplant, *CT*, computed tomography.

**FIGURE 2.**
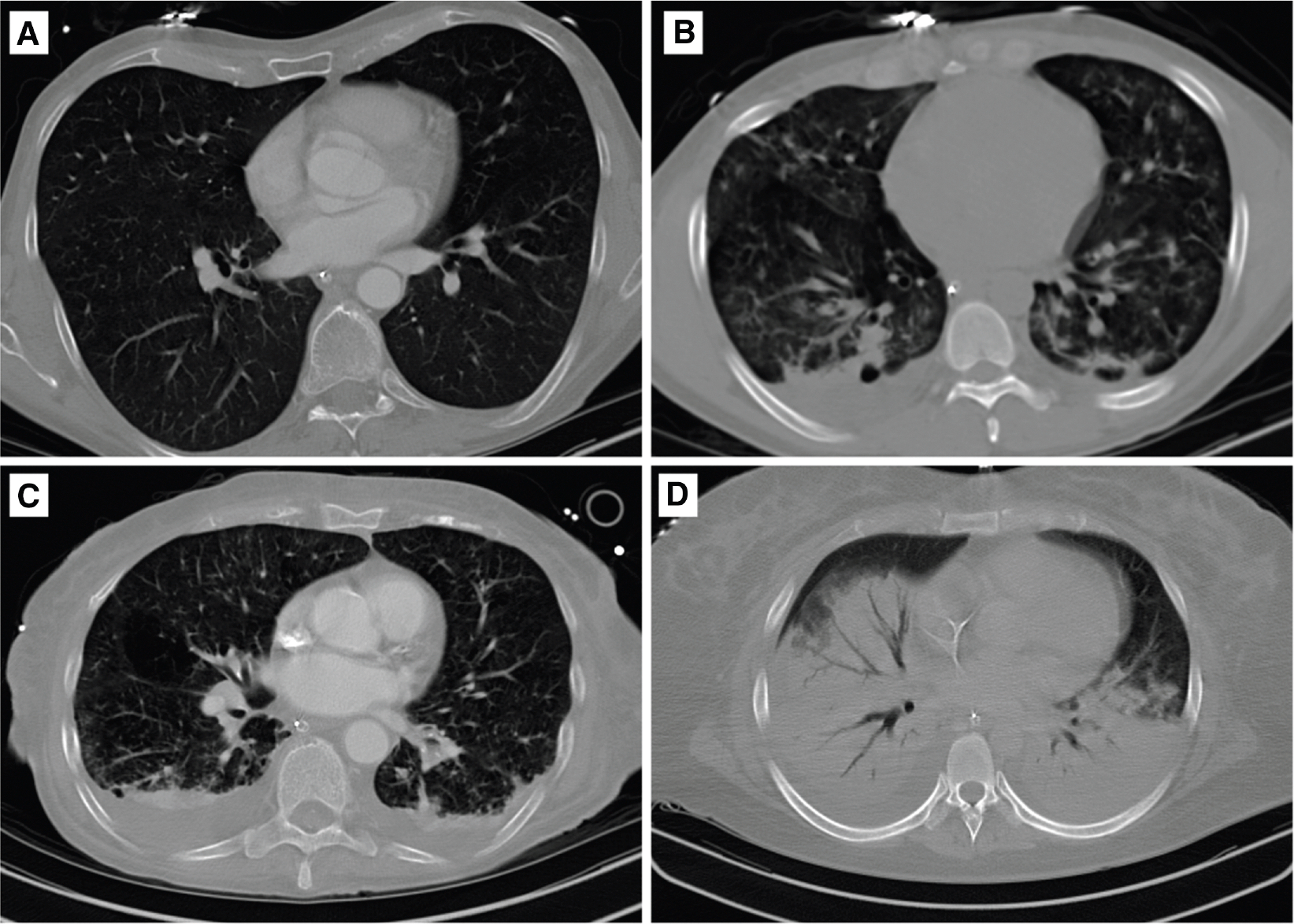
Representative computed tomography images from potential lung donors. A, No apparent abnormalities with lungs that were recovered. B, Interstitial lung disease with lungs not recovered. C, Emphysema with lungs not recovered. D, Traumatic lung contusions with lungs not recovered.

**FIGURE E1.**
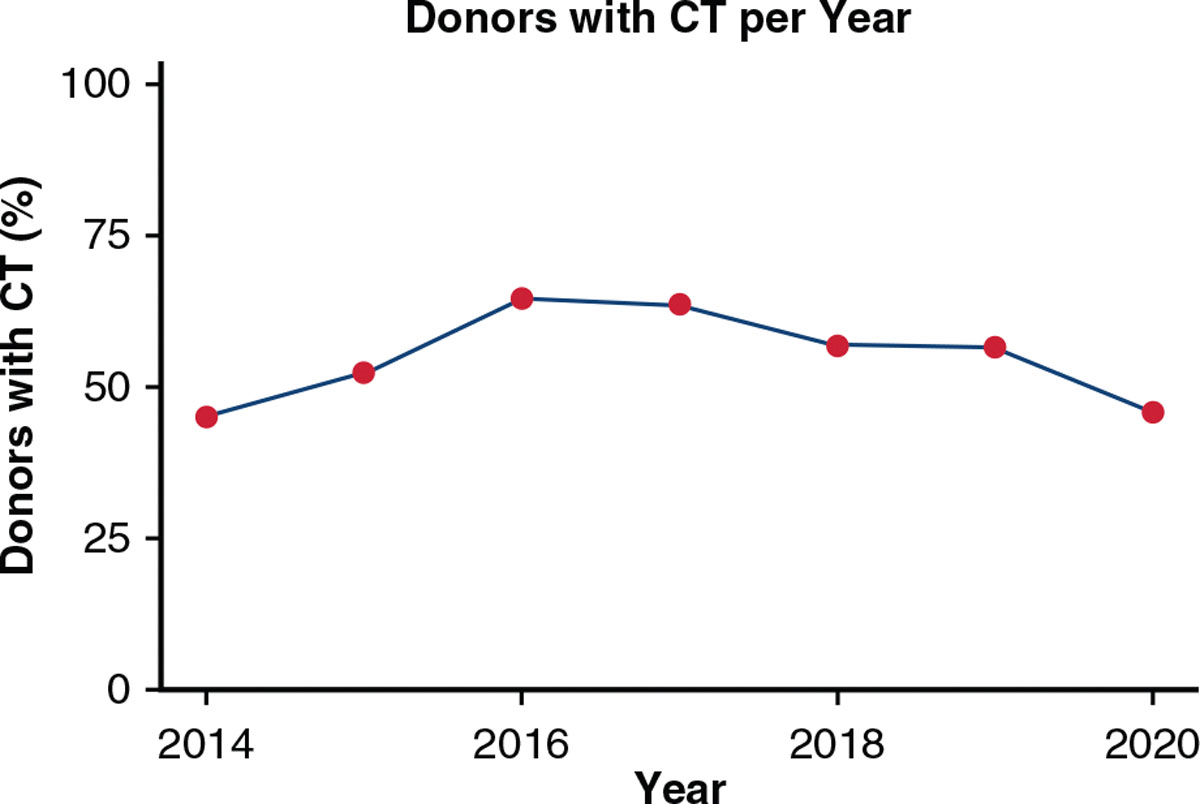
Plot of percentage of donors with a computed tomography (*CT*) as a function of time.

A total of 4854 brain-dead donors were managed at the 3 US OPOs during the study period. Of this cohort, 420 donors were excluded for age <18 years, and another 1980 donors were excluded due to a lack of CTs (Figure 1). The final study cohort was 2454 donors. Compared with donors without CTs, donors who underwent CTs tended to be younger, had a lower body mass index (BMI), were more likely to have a history of asthma or chronic obstructive pulmonary disease (COPD), were more frequently managed at specialized donor care facilities (SDCFs), and had a greater final partial pressure of arterial oxygen (Pao_2_) (Table E1). There was no association between a later year of donor management and likelihood donors having a CT (Figure E1). The lung acceptance rate in the cohort of donors who had CTs was 40.4%, whereas the acceptance rate of lungs in the cohort that did not have CTs was 22.3%. On univariable regression modeling lung acceptance, lung donors who underwent CT scans were 2.36 times (95% CI, 2.06–2.69) more likely to have lungs accepted for transplant. In the cohort with CTs, donors whose lungs were accepted tended to be younger, had lower BMI, and were less likely to have a history of smoking 20 pack-years or more, heavy alcohol use (≥2 drinks per day), previous myocardial infarction, asthma and/or COPD, or cancer (Table 1). In the multivariable logistic regression model including clinically relevant and statistically significant variables against lung acceptance (summarized in Table E2), CT evidence of emphysema (adjusted odds ratio [aOR], 0.31; 95% CI, 0.17–0.55), lobar or greater traumatic lung injury (aOR, 0.28; 95% CI, 0.11–0.64), and pulmonary edema (aOR, 0.61; 95% CI, 0.38–0.98) were associated with lungs not being accepted (Table 2). Representative CT images of potential lung donors with no apparent abnormalities, ILD, emphysema, and traumatic lung injury (lobar or greater) are shown in Figure 2.

**FIGURE 3.**
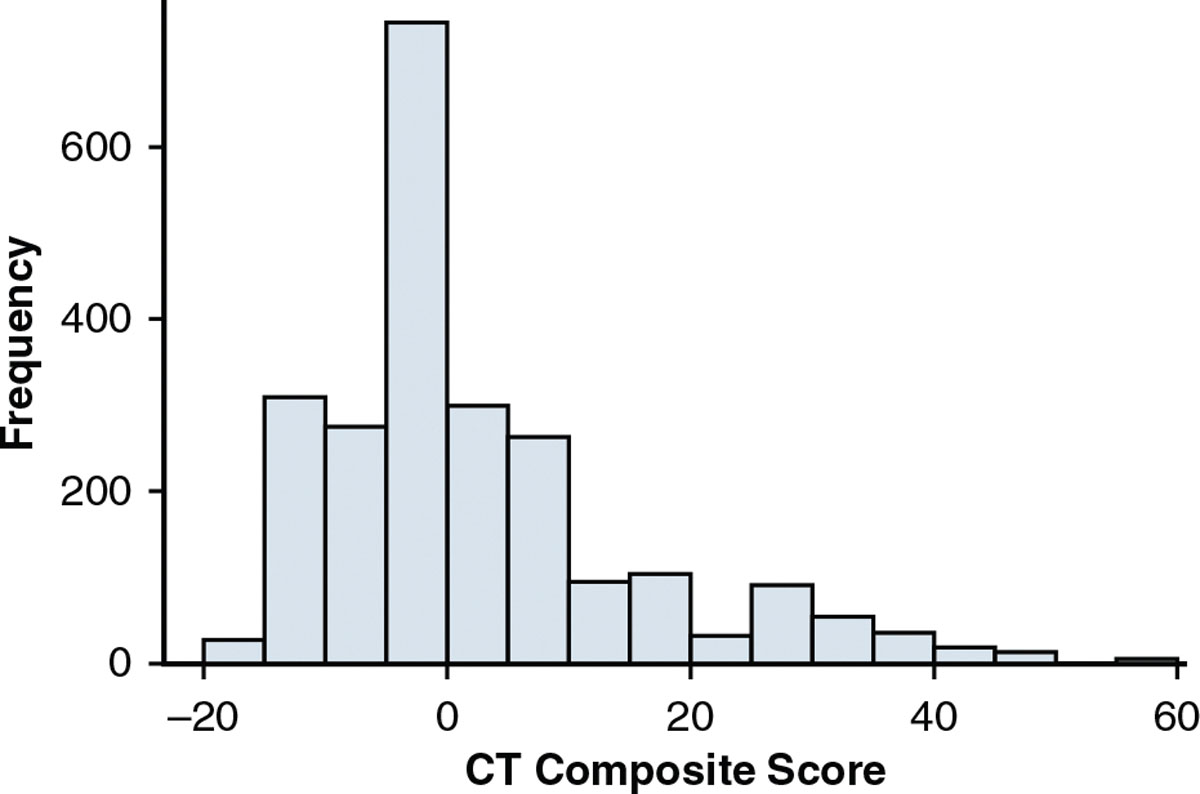
Histogram of computed tomography (*CT*) composite score among all brain-dead donors in final cohort.

The CT composite score ranged from −17.4 to 59.8, with median 0 and interquartile range −5.5 to 7.3 (Figure 3). Greater values represented more CT abnormalities. For example, 1 donor with evidence on CT of emphysema, lobar or greater atelectasis, moderate/large pleural effusion, ILD, and a Lung-RADS category 3 or greater nodule had a CT composite score of 48.73 and their lungs were not recovered, whereas another donor with CT evidence of only segmental or less atelectasis had a CT composite score of −13.32 and their lungs were recovered.

In both the CT and combined models, we found that every 1-unit increase in the CT composite score was associated with a ~4% lower likelihood of lung acceptance (*P* < .001). The beta coefficients for the CT composite score were −0.0406 and −0.0401 in CT model and combined model, respectively. The predictive abilities of the original CXR model, the CT model, and the combined model for lung acceptance in a given donor were comparable. The C-statistics of the CXR and CT models were 0.883 and 0.887, respectively, and the Brier scores were 0.135 and 0.134, respectively. In the combined model, the C-statistic was 0.890, and the Brier score was 0.132 (Table 3).

**FIGURE 4.**
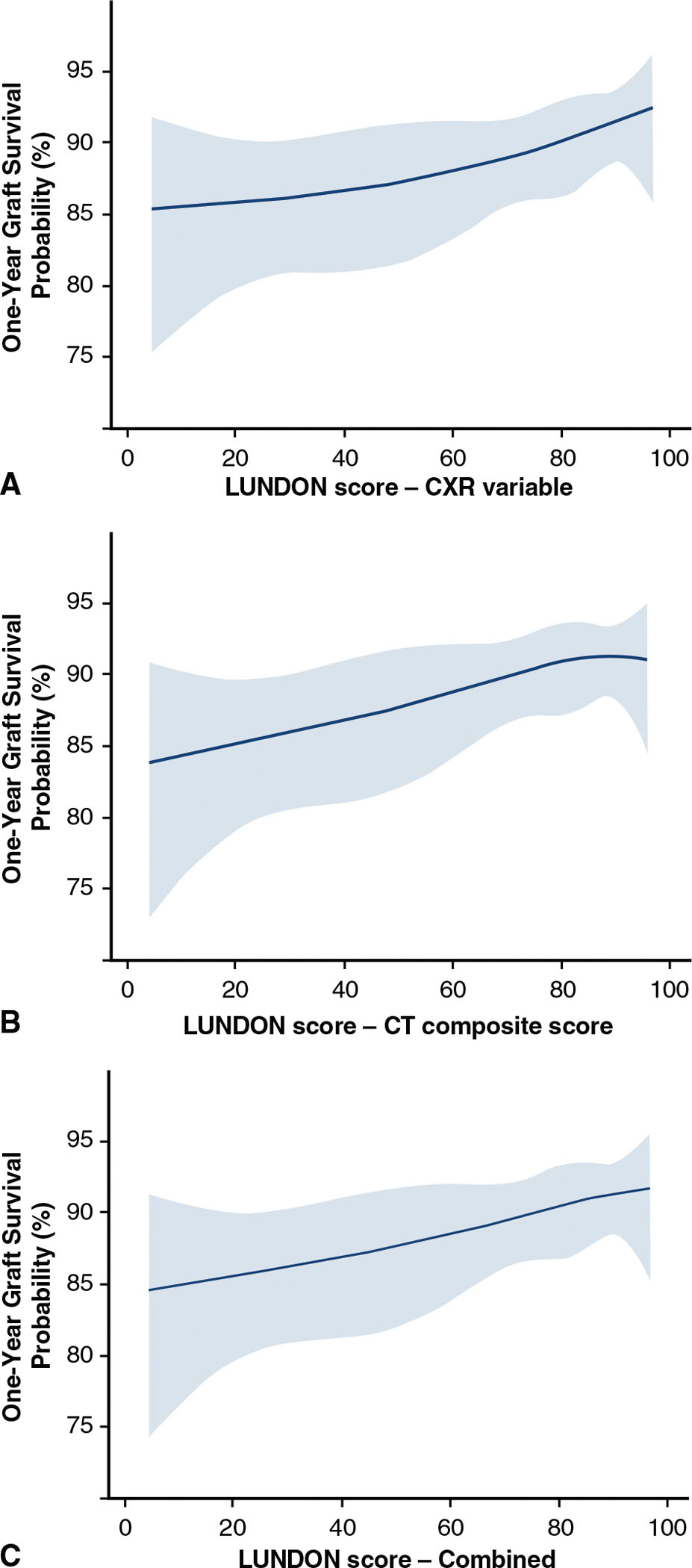
A, The RCS curve for the original LUNDON model with the CXR variable. B, The RCS curve for the LUNDON model with the CT composite score. C, The RCS curve for the LUNDON model with the CXR variable model and the CT composite score. *RCS*, Restricted cubic spline; *LUNDON*, Lung Donor; *CXR*, radiograph of the chest; *CT*, computed tomography.

A total of 944 recipients were matched with the donor cohort after exclusion of retransplants. We found a greater score was generally associated with a better probability of 1-year graft survival (Figure 4), with some minor differences between the 3 models. The RCS curve of the CXR model exhibited a consistently positive relationship between acceptance scores and 1-year graft survival probability (Figure 4, A). The RCS curve of the CT model demonstrated a consistently positive association between graft survival and scores below ~85 but plateaued for scores above this threshold (Figure 4, B). The RCS curve of the combined model resided between the RCS curves for the CXR and CT models (Figure 4, C).

## DISCUSSION

Our study developed a weighted CT composite score that was found to be independently associated with lung acceptance and tested the impact of its inclusion in the previously validated LUNDON model. Although we identified that specific CT characteristics are associated with lung acceptance, the addition of a CT composite score maintained but did not meaningfully improve the LUNDON model’s baseline excellent predictive ability of lung acceptance. Moreover, we found that all 3 iterations of the LUNDON model demonstrated comparable association with 1-year graft survival, albeit with minor differences.

In the cohort of donors who underwent CT imaging, we found on multivariable analysis that CT evidence of emphysema, lobar or greater traumatic lung injury, and pulmonary edema were negatively associated with lung acceptance. This is not surprising, because these findings are significantly linked to poor organ quality and adverse lung transplant outcomes. The presence of emphysema or edema on CT may be associated with an extensive donor smoking history, a factor that is also associated with declining a lung as the result of elevated risk for primary graft dysfunction and therefore earlier development of chronic lung allograft dysfunction.^20–22^ Pulmonary edema itself, which can develop in the setting of brain death as the result of a series of physiologic changes that heighten capillary permeability, leads to poor oxygenation and graft function in the acute postoperative setting.^23–25^ Severe traumatic lung injuries compromise the function of immune cells locally and systemically, and they may lead to increased bronchial mucus production and ventilator-associated pneumonia.^26,27^ Moreover, severe traumatic lung injury may indicate the donor has sustained significant polytrauma, a finding that is associated with massive blood transfusions that could lead to worse pulmonary function and prolonged mechanical ventilation postoperatively.^28^

In contrast, we observed that the presence of segmental or subsegmental atelectasis on CT was associated with lung acceptance. Rather than suggesting a protective effect from minor atelectasis, this finding likely reflects that dependent atelectasis is common among brain-dead donors who have often been supine and ventilated for days at a time.^2,29,30^ Furthermore, although minor atelectasis may lead to suboptimal oxygenation, this has little bearing on postoperative course and graft function, and unlike emphysema and traumatic lung injury, atelectasis is readily reversible with recruitment maneuvers and prone positioning.^30–34^

Although CT imaging provides a more accurate assessment of donor lung anatomy and pathology, there may be several reasons why the inclusion of a CT composite score did not significantly enhance the LUNDON model’s ability to predict lung acceptance. First, the LUNDON model already includes highly relevant clinical variables that have been shown to have an effect on lung acceptance. It is possible the additional granular information from a CT may not outweigh the impact of these clinical variables. Second, there is moderate concordance between CXR and CT findings in lung donors,^2^ which suggests that the additional information provided by CT imaging may only have a modest impact on the model’s performance. Third, some CT findings were readily modifiable (eg, atelectasis can be improved with recruitment maneuvers, pleural effusions may be drained, pneumonia may be treated with antibiotics, etc) and therefore may have had little impact on donor lung acceptance because they were improved or resolved with targeted donor management. Alternatively, modifiable abnormalities may not have been considered barriers to transplantation because they could be managed postengraftment. Finally, the original LUNDON model has excellent predictive strength at baseline, and additional parameters in the scoring system may only fine-tune rather than substantially improve its performance.

In our previous work, we identified a positive correlation between the original LUNDON model and 5-year graft survival.^5^ Here, we similarly demonstrated that greater scores in the CXR, CT, and combined models were associated with better 1-year graft survival, indicating that the CT composite score was also predictive of recipient outcomes. Although the differences between the curves were not substantial, we did note that the RCS curve for the CT model plateaued for LUNDON scores above ~85, indicating that there was a threshold at which increased scores did not translate into further improvements in survival. This finding may reflect the granular nature of the continuous CT variable, which was capable of capturing even minor abnormalities and further distinguishing between relatively normal lungs, whereas the binary CXR variable could not make finer distinctions between lungs with “normal” CXRs. The CT model’s enhanced sensitivity may have led to increased score variance among the highest quality lungs due to the detection of lower grade abnormalities, which ultimately had little effect on graft survival probability. The sensitivity of the CT model to minor imaging findings was likely mitigated by the CXR variable in the combined model, restoring a more consistently positive association between the LUNDON score and graft survival.

Notably, we found statistically significant differences between donors who underwent CTs and those who did not. Donors who underwent CTs were younger, had lower BMIs, had better final PaO_2_ values, and were more likely to donate lungs. Given the improved final PaO_2_ measurements among donors who had CTs, it is possible that subjecting a donor to a CT of the chest was a component of more aggressive donor management strategies, leading to its association with higher lung acceptance. Alternatively, there may have been selection bias because choosing to perform a CT scan in a donor may reflect an initial assessment that the individual is more likely to donate lungs. Given that donors with CTs were also more likely to have a history of asthma or COPD, it is possible that clinical indications could have motivated obtaining a CT for some donors. Importantly, we noted that donors who underwent CT imaging were more likely to be managed at SDCFs, suggesting that the availability of a dedicated CT scanner for donors or SDCF practice patterns may have influenced this decision in certain cases.

Although we did not observe a significant improvement in the LUNDON model’s predictive ability with the addition of a composite CT score, CT does offer greater insight into lung abnormalities that may not be present on CXR and may therefore enhance donor optimization efforts.^2^ This may be particularly valuable for improving the quality of donor lungs considered marginal. In light of the increasing adoption of SDCFs with on-site CT scanners, it is now also easier than before to obtain CT imaging of lung donors. Given the routine use of CT for abdominal organ imaging, the addition of a CT of the chest imposes little added cost or resource burden. Instituting standardized CT reporting would be a timely and relevant shift in donor management practices. Through its contribution to the development of larger datasets, the standardized reporting and categorization of donor CT findings on a national scale may facilitate future investigations of their impact on donor lung acceptance and their correlation with recipient outcomes. Future work with larger cohorts could assess whether a composite CT score has greater predictive ability of lung use in different population subsets, such as in donors with lower PaO_2_/inspired oxygen fraction ratios or older donors, or demonstrates an association with long-term recipient outcomes or quality of life.

This study has several key strengths, including robust methodology, the use of a modern and highly curated cohort, and high clinical relevance, given the increasing number of lung transplants being performed annually. However, this study is not without limitations. First, it is subject to the limitations of retrospective design and information bias. Second, our sample was limited to data from 3 participating OPOs from 2014 to 2020 with available donor CT information. Therefore, the findings of this study may not be generalizable to transplant programs nationally. In addition, the restricted sample size limited our ability to create a validation cohort, and we lacked data on longer-term outcomes, limiting our analysis for their association with donor CT findings. Third, we could only include the data from donors who underwent CT scans, which could have been performed selectively on the basis of clinical characteristics in some instances. In addition, the lack of available CT scanners at some facilities managing the donors may have been a logistical constraint that limited the number of donors who could undergo CT scans, as evidenced by our finding that management at an SDCF, which likely would have dedicated CT scanners for donors, was a major factor in whether a CT was obtained. Fourth, we acknowledge that the CTs and CXRs of the chest used in this study could have been obtained at different time points and resulted in some discrepancy between their respective findings for donors. However, we again note that there was a general concordance for both imaging methods, suggesting any timing differences did not result in significant differences in their findings.

## CONCLUSIONS

In summary, we developed a CT composite score associated with brain-dead donor lung acceptance from clinically relevant variables. We found the inclusion of the granular CT composite score in the LUNDON model maintained but did not meaningfully enhance its already-excellent predictive performance of lung acceptance. In addition, we found the incorporation of the CT composite score to the LUNDON model maintained its association with 1-year graft survival. This study invites an opportunity for the lung transplant community to reassess the optimal collection and reporting of radiologic criteria of donor lung evaluation.

## Supplementary Material

1 Webcast
You can watch a Webcast of this AATS meeting presentation by going to: https://www.aats.org/resources/the-addition-of-computed-tomog-9619.

2 Audio
Audio Recording: You can listen to the audio recording of the presentation and discussion associated with this paper: https://doi.org/10.1016/j.jtcvs.2026.01.035.

## Figures and Tables

**TABLE 1.** Comparison of baseline characteristics between donors who underwent CT scans with lungs accepted versus not accepted for transplantation

Variable	Lungs not accepted, n = 1463	Lungs accepted, n = 991	*P* value

Age, y	46 (33, 57)*	35 (26, 49)*	**<.001**
Male sex	829 (57%)	586 (59%)	.2
Race			
White	1043 (71%)	669 (68%)	ref
Black	300 (21%)	230 (23%)	.076
Hispanic	98 (6.7%)	74 (7.5%)	.3
Other	22 (1.5%)	18 (1.8%)	.4
Body mass index	29 (24, 34)*	26 (23, 30)*	**<.001**
Organ procurement organization			
ILIP	363 (54%)	315 (46%)	–
MIOP	501 (59%)	325 (41%)	–
MOMA	599 (63%)	351 (37%)	–
Smoking history (>20 pack-y)	452 (31%)	131 (13%)	**<.001**
Heavy alcohol use (≥2 drinks/d)	376 (26%)	216 (22%)	**.027**
Intravenous drug use	354 (24%)	223 (23%)	.3
History of myocardial infarction	176 (12%)	38 (3.8%)	**<.001**
Asthma and/or COPD	625 (43%)	322 (32%)	**<.001**
Chronic kidney disease	434 (30%)	283 (29%)	.6
History of cancer	79 (5.4%)	24 (2.4%)	**<.001**

*P* values less than or equal to .05 were statistically significant and bolded in the table. *CT*, Computed tomography; *ILIP*, Gift of Hope Organ & Tissue Donor Network; *MIOP*, Gift of Life Michigan; *MOMA*, Mid-America Transplant; *COPD*, chronic obstructive pulmonary disease.

* Median (interquartile range).

**TABLE 2.** Various findings from CT scan of the chest associated with lung acceptance for transplantation on multivariable logistic regression analysis

Finding on CT of the chest	Lungs not accepted, n = 1463	Lungs accepted, n = 991	Adjusted odds ratio	*P* value*

Pulmonary hypertension and/or right heart strain	114 (7.8%)	27 (2.7%)	0.68	.3
Emphysema	205 (14%)	37 (3.7%)	**0.31**	**<.001**
Interstitial lung disease	23 (1.6%)	5 (0.5%)	0.63	.6
Traumatic lung injury				
None	1354 (93%)	908 (92%)		
Segmental or less	64 (4.4%)	60 (6.1%)	1.18	.6
Lobar or greater	45 (3.1%)	23 (2.3%)	**0.28**	**.003**
Atelectasis				
None	549 (38%)	362 (37%)		
Segmental or less	490 (33%)	416 (42%)	**1.73**	**<.001**
Lobar or greater	424 (29%)	213 (21%)	1.1	.012
Pneumonia				
None	931 (64%)	688 (69%)		
Segmental or less	300 (21%)	180 (18%)	0.86	.4
Lobar or greater	232 (16%)	123 (21%)	0.73	.083
Pulmonary embolism	56 (4%)	19 (2%)	0.96	>.9
Pleural effusion				
None	1098 (75%)	785 (79%)		
Small	240 (16%)	132 (13%)	0.86	.4
Moderate or large	125 (8.5%)	74 (7.5%)	0.97	.9
Pulmonary edema	148 (10%)	61 (6.2%)	**0.61**	**.044**
Nodules Lung-RADS category 3 or greater	106 (7.2%)	42 (4.2%)	0.65	.14

Covariates included in this model are summarized in Table E2. Bold text indicates statistically significant adjusted odds ratios and *P* values. *CT*, Computed tomography; *Lung-RADS*, Lung CT Screening Reporting & Data System.

* *P* value represents multivariable logistic regression model, with less than or equal to .05 considered statistically significant.

**TABLE 3.** Comparison of the CXR, CT, and combined models’ predictive ability of lung acceptance in a given donor

	CT composite score				
Variable	aOR	95% CI	*P* value	C-statistic	Brier score

CXR model	–	–	–	0.883	0.135
CT model	0.96	0.95–0.97	<.001	0.887	0.134
Combined model	0.96	0.95–0.97	<.001	0.890	0.132

*CXR*, Radiograph of the chest; *CT*, computed tomography; *aOR*, adjusted odds ratio.

**TABLE E1.** Baseline characteristics of donors with CTs versus donors without CTs

Variable	CT, n = 2454	No CT, n = 1980	*P* value

Age, y	41 (30, 54)*	49 (34, 58)*	**<.001**
Male sex	1415 (58%)	1158 (58%)	.6
Race			**<.001**
White	1712 (70%)	1187 (60%)	
Black	530 (22%)	530 (27%)	
Hispanic	172 (7.0%)	208 (11%)	
Other	40 (1.6)	55 (2.8%)	
Body mass index, kg/m^2^	27 (24, 32)*	29 (24, 34)*	**<.001**
Organ procurement organization			
ILIP	678 (28%)	1356 (68%)	–
MIOP	826 (34%)	614 (31%)	–
MOMA	950 (39%)	10 (0.01%)	–
SDCF	1339 (55%)	208 (10.5%)	**<.001**
Death by asphyxiation or drowning	139 (5.7%)	85 (4.3%)	**.038**
Smoking history (>20 pack-y)	584 (24%)	451 (23%)	.4
Heavy alcohol use (≥2 drinks/d)	592 (24%)	382 (19%)	**<.001**
Intravenous drug use	577 (24%)	500 (25%)	.2
Bloodstream infection present	244 (10%)	219 (11%)	.2
History of myocardial infarction	214 (8.7%)	221 (11%)	**.007**
Cardiac arrest after death	429 (17%)	347 (18%)	1
Asthma and/or COPD	947 (39%)	655 (33%)	**<.001**
Abnormal initial CXR	1504 (62%)	1243 (64%)	.3
Initial PaO_2_, mm Hg	163 (97, 287)*	162 (96, 302)*	.8
PaO_2_ prior to harvest, mm Hg	288 (126, 435)*	156 (97.8, 335)*	**<.001**
Chronic kidney disease	717 (29%)	515 (26%)	**.018**
Creatinine, mg/dL	1.52 (1.15, 2.35)*	1.7 (1.21, 2.93)*	**<.001**
History of cancer	103 (4.2%)	95 (4.8%)	.3

*CT*, Computed tomography; *ILIP*, Gift of Hope Organ & Tissue Donor Network; *MIOP*, Gift of Life Michigan; *MOMA*, Mid-America Transplant; *SDCF*, specialized donor care facility; *COPD*, chronic obstructive pulmonary disease; *CXR*, radiograph of the chest; *PaO_2_*, partial pressure of oxygen in arterial blood.

* Median (interquartile range). *P* values less than or equal to .05 were statistically significant and bolded in the table.

**TABLE E2.** Beta coefficients of multivariable logistic regression model used to create weighted CT composite score

Variable	Beta coefficient	Adjusted odds ratio	95% CI	*P* value

Age	−0.0171	0.98	0.97–0.99	**.001**
Body mass index, kg/m^2^	−0.0035	1	0.98–1.02	.7
Death by asphyxiation or drowning	−0.0876	0.92	0.52–1.63	.8
Creatinine, mg/dL	−0.0382	0.96	0.88–1.05	.4
PaO_2_ prior to harvest, mm Hg	0.0098	1.01	1.01–1.01	**<.001**
Cardiac arrest after death	0.5020	1.65	1.09–2.51	**.018**
Bloodstream infection present	−0.0587	0.94	0.62–1.43	.8
Smoking history (>20 pack-y)	−0.4342	0.65	0.45–0.92	**.017**
History of myocardial infarction	−0.6537	0.52	0.29–0.91	**.024**
Heavy alcohol use (≥2 drinks/d)	0.1115	1.12	0.82–1.52	.5
Asthma and/or COPD	0.0088	1.01	0.73–1.4	>.9
History of cancer	−0.4161	0.66	0.32–1.34	.3
HCV seropositivity	−1.0715	0.34	0.19–0.60	**<.001**
CMV seropositivity	−0.0362	0.96	0.75–1.25	.8
Acute renal failure	−0.2000	0.82	0.46–1.46	.5
T4 use	−0.8274	0.44	0.31–0.60	**<.001**
Inotrope use	1.1539	3.17	2.14–4.74	**<.001**
Blood transfusions (none)	–	–	–	
Blood transfusions (1–5 units)	0.1204	1.13	0.85–1.50	.4
Blood transfusions (6–10 units)	−0.1470	0.86	0.54–1.38	.5
Blood transfusions (>10 units)	−0.2107	0.81	0.43–1.55	.5
Lung infection present	−0.0547	0.95	0.67–1.34	.8
Antibiotic use	−0.4011	1.49	1.08–2.07	**.016**
Number of bronchoscopies performed	0.1586	1.17	1.05–1.31	**.005**
Significant bronchoscopic abnormality*	−0.5524	0.58	0.43–0.77	**<.001**
CT – pulmonary hypertension or RHS	−0.3808	0.68	0.35–1.32	.3
CT – emphysema	−1.1638	0.31	0.17–0.55	**<.001**
CT – interstitial lung disease	−0.4651	0.63	0.10–2.98	.6
CT – TLI (none)	–	–	–	
CT – TLI (segmental or less)	0.1683	1.18	0.66–2.13	.6
CT – TLI (lobar or greater)	−1.2896	0.28	0.11–0.64	**.003**
CT – atelectasis (none)	–	–	–	
CT – atelectasis (segmental or less)	0.5469	1.73	1.27–2.36	**<.001**
CT – atelectasis (lobar or greater)	0.0935	1.1	0.78–1.55	.6
CT – pneumonia (none)	–	–	–	
CT – pneumonia (segmental or less)	−0.1509	0.86	0.62–1.2	.4
CT – pneumonia (lobar or greater)	−0.3215	0.73	0.5–1.04	.083
CT – pulmonary embolism	−0.0423	0.96	0.44–2.04	>.9
CT – pleural effusion (none)	–	–	–	
CT – pleural effusion (small)	−0.1471	0.86	0.59–1.25	.4
CT – pleural effusion (moderate/large)	−0.0323	0.97	0.58–1.60	.9
CT – pulmonary edema	−0.4875	0.61	0.38–0.98	**.044**
CT – nodule Lung-RADS category 3 or greater	−0.4332	0.65	0.36–1.16	.14

Model intercept: −2.5037. *CT*, Computed tomography; *PaO2*, partial pressure of oxygen in arterial blood; *COPD*, chronic obstructive pulmonary disease; *HCV*, hepatitis C virus; *CMV*, cytomegalovirus; *T4*, thyroxine; *RHS*, right heart strain; *TLI*, traumatic lung injury; *Lung-RADS*, Lung CT Screening and Reporting & Data System.

* Significant bronchoscopic abnormality includes evidence of aspiration, purulence, and/or repooling secretions. *P* values less than or equal to .05 were statistically significant and bolded in the table.

## Box

**BOX 1.** Derivation of an equation to calculate the CT composite score

Finding on CT scan	Variable name in equation below	*β*	Weight (= *β*/−4.1054)

Pulmonary hypertension or right heart strain	pHTN or RHS	−0.3808	9.28%
Emphysema	Emphysema	−1.1638	28.35%
Interstitial lung disease	ILD	−0.4651	11.33%
Traumatic lung injury	–	–	–
Segmental or less	TLI_segmental or less_	0.1683	−4.10%
Lobar or greater	TLI_lobar or greater_	−1.2896	31.41%
Atelectasis	–	–	–
Segmental or less	Atelectasis_segmental or less_	0.5469	−13.32%
Lobar or greater	Atelectasis_lobar or greater_	0.0935	−2.28%
Pneumonia	–	–	–
Segmental or less	PNA_segmental or less_	−0.1509	3.68%
Lobar or greater	PNA_lobar or greater_	−0.3215	7.83%
Pulmonary embolism	PE	−0.0423	1.03%
Pleural effusion	–	–	–
Small	Effusion_small_	−0.1471	3.58%
Moderate or large	Effusion_moderate or large_	−0.0323	0.79%
Pulmonary edema	Edema	−0.4875	11.87%
Nodule ≥ Lung-RADS3	Nod3	−0.4332	10.55%
	Total	−4.1054	100%

CT composite score = 9.28 × [pHTN or RHS] + 28.35 × [Emphysema] + 11.33 × [ILD] − 4.1 × [TLI_segmental or less_] + 31.41 × [TLI_lobar or greater_] − 13.32 × [Atelectasis_segmental or less_] − 2.28 × [Atelectasis_lobar or greater_] + 3.68 × [PNA_segmental or less_] + 7.83 × [PNA_lobar or greater_] + 1.03 × [PE] + 3.58 × [Effusion_small_] + 0.79 × [Effusion_moderate or large_] + 11.87 × [Edema] + 10.55 × [Nod3].

Beta coefficients of individual CT findings (*β*) from amultivariable logistic regression model of lung acceptance were summed and each assigned a weight on the basis of this sum (Weight). The CT composite score for a lung donor was then calculated using the equation shown in the box, based on the calculated weights and presence or absence of each CT finding for that donor.

*CT*, Computed tomography.

## PERSPECTIVE

Findings on CT of the chest may influence lung use and predict 1-year graft survival. Adopting standardized reporting of CT findings to the Scientific Registry of Transplant Recipients would be a timely shift in donor management practices and facilitate future, larger-scale investigations into their impact on lung acceptance and lung transplant outcomes.

## Abbreviations and Acronyms

aOR: adjusted odds ratio
BMI: body mass index
COPD: chronic obstructive pulmonary disease
CT: computed tomography
CXR: radiograph of the chest
ILD: interstitial lung disease
LUNDON: Lung Donor
Lung-RADS: Lung CT Screening Reporting & Data System
OPO: organ procurement organization
Pao_2_: partial pressure of oxygen in arterial blood
RCS: restricted cubic spline
SDCF: specialized donor care facility
SRTR: Scientific Registry of Transplant Recipients
